# Primary fallopian tube carcinoma: review of MR imaging findings

**DOI:** 10.1007/s13244-015-0416-y

**Published:** 2015-07-07

**Authors:** Filipe Veloso Gomes, João Lopes Dias, Rita Lucas, Teresa Margarida Cunha

**Affiliations:** Centro Hospitalar do Algarve, Faro, Portugal; Department of Biomedical Sciences and Medicine, Regenerative Medicine Program, University of Algarve, Faro, Portugal; Department of Radiology, Hospital de S.José, CHLC, Lisbon, Portugal; Nova Medical School/Faculdade de Ciências Médicas, Lisbon, Portugal; Department of Radiology, Hospital de Sto. António dos Capuchos, CHLC, Lisbon, Portugal; Department of Radiology, Instituto Português de Oncologia de Lisboa Francisco Gentil, Lisbon, Portugal

**Keywords:** Fallopian tube neoplasms, Magnetic resonance imaging, High-grade serous carcinoma, Epithelial ovarian cancer, Peritoneal cancer

## Abstract

**Objectives:**

To review the epidemiological and clinical features of primary fallopian tube carcinoma (PFTC), and to illustrate the spectrum of MRI findings, with pathological confirmation.

**Methods:**

This article reviews the relevant literature on the epidemiological, clinical, and imaging features of primary fallopian tube carcinoma, with pathological confirmation, using illustrations from the authors’ teaching files.

**Results:**

Primary fallopian tube carcinoma came under focus over the last few years due to its possible role on the pathogenesis of high-grade serous epithelial ovarian and peritoneal cancers. Typical symptoms, together with the presence of some of the most characteristic MRI signs, such as a “sausage-shaped” pelvic mass, hydrosalpinx, and hydrometra, may signal the presence of primary fallopian cancer, and allow the radiologist to report it as a differential diagnosis.

**Conclusions:**

Primary fallopian tube carcinoma has a constellation of clinical symptoms and magnetic resonance imaging features, which may be diagnostic. Although these findings are not present together in the majority of cases, radiologists who are aware of them may include the diagnosis of primary fallopian tube cancer in their report more frequently and with more confidence.

***Teaching Points*:**

• *PFTC may be more frequent than previously thought*

• *PFTC has specific clinical and MRI characteristics*

• *Knowledge of typical PFTC signs enables its inclusion in the differential diagnosis*

• *PFTC is currently staged under the 2013 FIGO system*

• *PFTC is staged collectively with ovarian and peritoneal neoplasms*

## Introduction

Primary fallopian tube carcinoma (PFTC) has been described as one of the rarest malignancies of the female genital tract, accounting for around 1 % of all gynaecologic malignancies, occurring predominantly in post-menopausal women at a mean age of 55 years [1–7].

PFTC has come under focus in recent years, particularly in pathology and oncology scientific literature, given the likely role on the pathogenesis of ovarian cancer. This may have led to underestimate the true incidence of PFTC as ovarian cancer in the past, with significant clinical impact on the management of these patients [1, 8–12]. Several papers have emerged supporting the theory that high-grade serous ovarian carcinoma, as well as peritoneal carcinoma, may in fact originate from occult high-grade serous carcinoma in the fallopian tubes. The fimbriated ends of fallopian tubes could be the dominant site of origin, particularly in high-risk BRCA mutation carriers [7, 9, 11, 13–19].

PFTC is usually not suspected pre-operatively or even intra-operatively due to its nonspecific clinical and surgical presentation, particularly when disseminated [1, 3, 20–22]. Regarding imaging studies, PFTC is also rarely diagnosed in pre-operative studies due to nonspecific findings and overlap with ovarian cancer features [2, 3, 21–27].

The purpose of this study is to review the epidemiological and clinical features of PFTC, and to illustrate the spectrum of MRI findings, with pathological correlation.

## Epidemiology

PFTC is considered a rare and aggressive type of tumour, representing 0.14–1.8 % of the total gynaecological malignancies [1, 5, 28]. The incidence of PFTC may be on the rise for reasons that have not been completely understood. Associations between PFTC and socio-economic status and occupation have been described, with women of higher social classes and education being at greater risk [5, 29].

## Clinical presentation

The aetiology of this type of tumour has not been completely explained. Hormonal, reproductive, and genetic factors may play a role, along with the presence of chronic inflammation of the pelvis [28].

PFTC may have a constellation of characteristic symptoms, namely colicky abdominal or pelvic pain and adnexal mass, relieved by intermittent, profuse, serosanguineous vaginal discharge, which constitute Laztko’s triad (seen in only 15 % of patients). *Hydrops tubae profluens* is a syndrome characterized by the relief of pain and shrinkage of the abdominal or pelvic mass by a vaginal discharge, which is caused by filling and emptying of a sub-occluded fallopian tube (seen in only 5 % of patients). Typical symptoms, however, occur in only a minority of patients with PFTC, and most women present with less specific symptoms at the time of diagnosis [1, 28, 30–33]. The age of presentation is commonly between 40 and 60 years, with a mean age of 55 years [28]. The pre-operative diagnosis of PFTC is rarely performed, with clinical signs and symptoms pointing towards the more frequently occurring ovarian cancer or pelvic inflammatory disease. Tumour markers, particularly CA-125, have no role in the diagnosis of PFTC. Elevated CA-125 levels are, nevertheless, indicative of poor prognosis, and can be used during follow-up, as a marker of disease recurrence [34].

PFTC should be considered in the differential diagnosis of patients with postmenopausal bleeding with negative diagnostic curettage, cervical smear with intermittent suspicious abnormalities, and unexplained persistent vaginal discharge [28, 30].

## Pathological diagnosis

Pathology remains the mainstay for diagnosis of PFTC. Serous carcinoma of the fallopian tube is the most common histological type of tubal carcinoma. It is an invasive tumour growing in papillary, glandular, and solid patterns with high grade nuclear atypia. These tumours are identical to their ovarian counterparts.

The second most common type of tumour is the endometrioid carcinoma, followed by undifferentiated, clear cell, mucinous, and transitional carcinomas [35].

PFTC is graded according to its differentiation and extent of solid components, with most tumours being poorly differentiated [4, 36].

Initial diagnostic criteria for the diagnosis of PFTC were proposed by Hu et al. in 1950 [37]. These were revised later by Sedlis et al. [38, 39] and currently include the following: 1. The main tumour arises from the endosalpinx; 2. The histological pattern reproduces the epithelium of the tubal mucosa; 3. The transition from benign to malignant tubal epithelium is demonstrable; 4. The ovaries or endometrium are either normal or contain a tumour that is smaller than the tumour in the tube [28, 31]. Recently, Singh et al. [40], proposed an approach to the pathological assignment of primary site in high-grade serous tubal, ovarian, and peritoneal carcinoma. According to Singh, the identification of tumour inside the fallopian tubes, even in the presence of larger tumours in other localizations, supports the diagnosis of PFTC [40].

Dissemination of PFTC occurs through the transcoelomic route with implantation of cells throughout the abdominal cavity, similarly to ovarian cancer, and also through continuity to adjacent organs, transluminal migration, haematogenous, and lymphatic spread. Distant metastases are more common in PFTC than ovarian cancer [28, 36, 41], but a biopsy of such lesions would not distinguish them with certainty [41].

## Staging

The currently accepted staging system for PFTC was developed by the International Federation of Gynecology and Obstetrics (FIGO), and currently PFTC, ovarian, and peritoneal cancers are staged collectively within the same system, although occasionally it may be impossible to attribute the tumour to a primary site [42]. Table 1 outlines the 2013 FIGO staging classification for cancer of the ovary, fallopian tube, and peritoneum.

**Table 1:** 2013 FIGO staging classification for cancer of the ovary, fallopian tube, and peritoneum

Primary tumor (T)		
TNM	FIGO	Description
Tx	–	Primary tumor cannot be assessed
T0	–	No evidence of primary tumor
T1	I	Tumor confined to the ovaries or fallopian tubes
T1a	IA	Tumor limited to one ovary (capsule intact) or fallopian tube; no tumor on ovarian or fallopian tube surface; no malignant cells in ascites or peritoneal washings
T1b	IB	Tumor limited to both ovaries (capsule intact) or fallopian tubes; no tumor on ovarian or fallopian tube surface; no malignant cells in ascites or peritoneal washing
T1c	IC	Tumor limited to one or both ovaries or fallopian tubes with any of the following:
	IC1	- Surgical spill
	IC2	- Capsule ruptured before surgery or tumor on ovarian or fallopian tube surface
	IC3	- Malignant cells in the ascites or peritoneal washings
T2	II	Tumor involves one or both ovaries or fallopian tubes with pelvic extension (below pelvic brim) or peritoneal cancer
T2a	IIA	Extension and/or implants on uterus and/or tube(s) and/or ovaries
T2b	IIB	Extension to other pelvic intraperitoneal tissues
T3	III	Tumor involves one or both ovaries or fallopian tubes, or peritoneal cancer, with cytologically or histologically confirmed spread to the peritoneum outside the pelvis and/or metastases to the retroperitoneal lymph nodes
T3a	IIIA	Positive retroperitoneal lymph nodes and/or microscopic metastasis beyond pelvis
	IIIA1	Positive retroperitoneal lymph nodes only (cytologically or histologically proven)
	IIIA1 (i)	Metastasis up to 10 mm in greatest dimension
	IIIA1 (ii)	Metastasis more than 10 mm in greatest dimension
	IIIA2	Microscopic extrapelvic (above the pelvic brim) peritoneal involvement, with or without positive retroperitoneal lymph nodes
T3b	IIIB	Macroscopic peritoneal metastasis beyond pelvis up to 2 cm in greatest dimension, with or without metastasis to the retroperitoneal lymph nodes
T3c	IIIC	Macroscopic peritoneal metastasis beyond the pelvis more than 2 cm in greatest dimension, with or without metastasis to the retroperitoneal lymph nodes (includes extension of tumor to capsule of liver and spleen without parenchymal involvement of either organ)
	IV	Distant metastasis excluding peritoneal metastases
	IVA	Pleural effusion with positive cytology
	IVB	Parenchymal metastases and metastases to extra-abdominal organs (including inguinal lymph nodes and lymph nodes outside the abdominal cavity)
Regional lymph nodes (N)		
Nx	–	Regional lymph nodes cannot be assessed
N0	–	No regional lymph node metastases
N1	III	Regional lymph node metastases
Distant metastasis (M)		
M0	–	No distant metastases
M1	IV	Distant metastasis (excludes peritoneal metastases)
	IVA	Pleural effusion with positive cytology
	IVB	Parenchymal metastasis and metastases to extra-abdominal organs (including inguinal lymph nodes and lymph nodes outside the abdominal cavity)#

Adapted from Prat J (2014) Staging Classification for Cancer of the Ovary, Fallopian Tube, and Peritoneum. Int J Gynaecol Obstet 124:1–5 (reference 42). (*) Dense adhesions with histologically proven tumour cells justify upgrading to stage II. (#) Transmural bowel infiltration or umbilical deposit are stage IVB

## Treatment

Current management of PFTC follows the same guidelines as ovarian cancer in terms of surgical staging, debulking, and adjuvant chemotherapy [43]. The main goal is to decrease tumour load surgically. The surgical approach consists of total abdominal hysterectomy, bilateral salpingo-oophorectomy, and infra-colic omentectomy, appendicectomy, peritoneal washings, and peritoneal biopsies [43, 44].

Routine pelvic and para-aortic lymphadenectomy is considered to be essential by some authors due to the strong likelihood of lymphatic spread. However, this issue remains controversial, and others believe that retroperitoneal node sampling and dissection suffices [28, 45].

Postoperatively, chemotherapy plays an important role in the management of early-stage PFTC, usually with platinum-based combination chemotherapy. The efficacy of current chemotherapy regimens has led to the abandonment of radiotherapy as a treatment option for PFTC due to its poor results and serious complications [1, 28].

Hormonal therapies may be of value in the future, given the sensitivity and response of the fallopian tube epithelium to hormonal fluctuations, although there are no current recommendations [1, 28, 45].

## Prognosis

The main prognostic factors identified for increased survival include stage, age, and residual tumour after surgery, serous subtype, and elevated pre-treatment CA-125 [28, 36]. The presence of specific symptoms may lead to an earlier diagnosis and, consequently, improved survival.

Serous PFTC in advanced stage may have a better survival than its ovarian or peritoneal counterparts [43, 46]. Earlier stage PFTC disease could also have better survival rates, given the possibility of presenting earlier and the established role of lymphadenectomy in its management. Other authors have suggested that survival outcomes are similar between PFTC and ovarian cancer, in support of identical therapeutic approaches for both types of tumours [47], and some report better survival in ovarian cancer patients compared to equivalent stage patients with PFTC. The 5-year survival rate of PFTC ranges between 22 and 57 % [28].

Recurrent disease, which occurs on average 2 to 3 years after initial treatment, is associated with a dismal prognosis due to the lack of alternative treatments available [28].

## Imaging PFTC

Imaging diagnosis of PFTC is important because it can help planning adequate initial surgery and avoid second-look laparotomy. Imaging findings of PFTC are usually nonspecific, and a tubo-ovarian abscess or ovarian tumour may appear to be the most likely diagnosis given their higher prevalence [26].

Similarly to other gynaecologic tumours, the imaging approach includes ultrasound as the initial modality, followed by MRI for undetermined or suspicious adnexal masses and computed tomography (CT) or MRI for complete staging. CT is only used for staging purposes and not for pelvic mass characterization due to low soft-tissue contrast in comparison to MRI [2, 48].

On greyscale ultrasound, PFTC may be suspected in the presence of a tubular-shaped mass (or sausage-shaped) or a lobular mass with a cogwheel pattern [49]. On Doppler ultrasound, a low-impedance flow within the solid components may be a clue [50]. Variability of imaging characteristics in serial imaging may also point towards PFTC [51].

MRI is the modality of choice for evaluating an undetermined pelvic mass on ultrasound and also to evaluate the local burden of tumour [52, 53]. Recently Ma et al. [48] have outlined the use of MRI for differentiating PFTC from epithelial ovarian cancer (EOC). According to their assessment of MRI features, the characteristic appearance of PFTC was a relatively small, tubular-shaped (or sausage-shaped) mass, with homogenous signal, low signal intensity on T1 weighted images (T1WI), high signal intensity on T2 weighted images (T2WI), mild to moderate enhancement, and hydrosalpinx or intrauterine fluid. Ma et al. identified tubular (sausage) shape, hydrosalpinx, and the presence of intra-uterine fluid as the most specific direct and indirect signs of PFTC. The combination of an adnexal mass with at least one of the former features yields a high diagnostic accuracy [48].

### Anatomy of the fallopian tubes on MRI

**Fig. 1:**
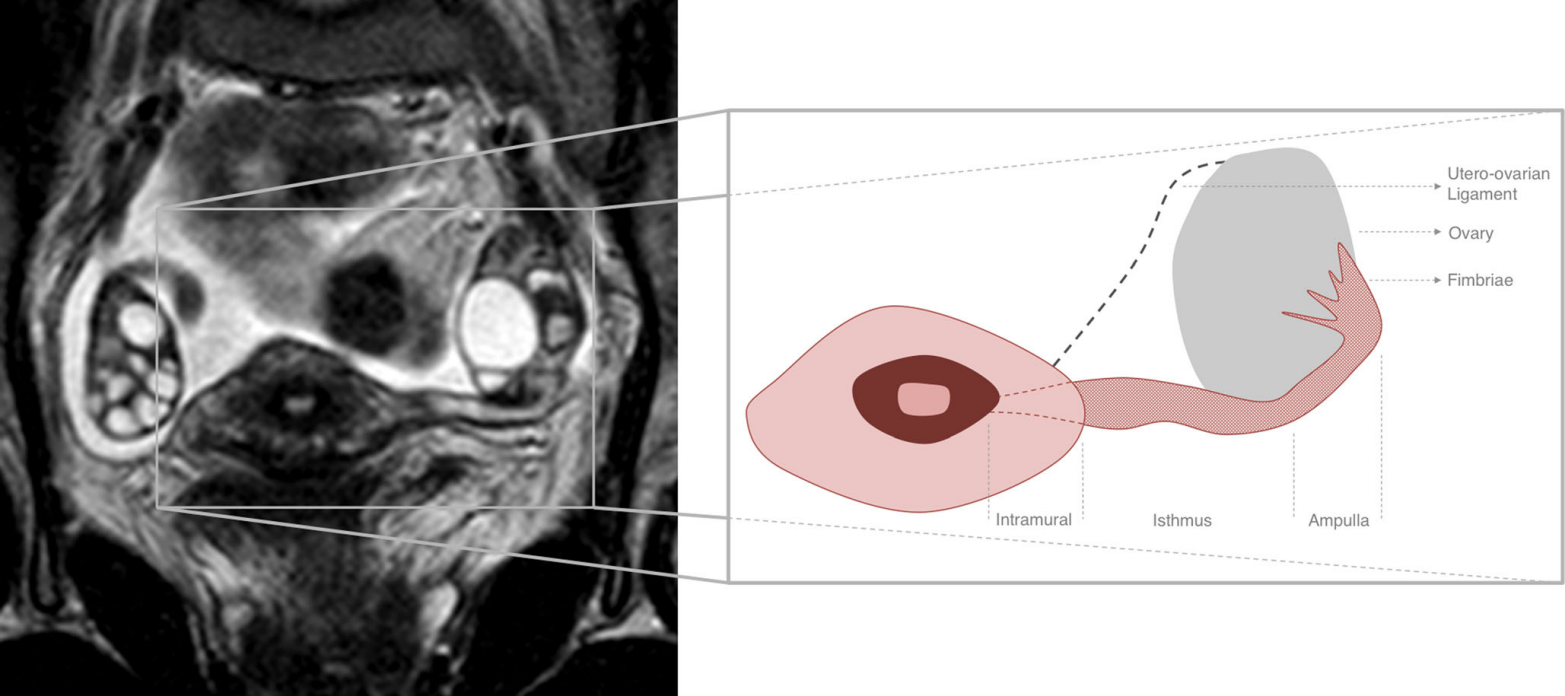
Thirty-two-year-old female imaged for other purposes. T2WI coronal oblique section through the pelvis showing a normal uterus, both ovaries, and the full length of the left fallopian tube. On the right hand side of the image, an illustration of the fallopian tube anatomy can be observed, based on the MR image see on the left, showing the four segments of the tube: intramural, isthmus, ampulla, and fimbriae

The normal fallopian tubes are usually not visualized on pelvic MRI. In the presence of intraperitoneal fluid, they may be seen as paired thin structures, extending from the ovaries to the uterine cornua, in the superior edge of the broad ligament [2]. They extend for about 10–12 cm although they will appear shorter due to being convoluted on cross-sectional imaging. The fallopian tubes are divided into four portions, the intramural/interstitial on the medial end, the isthmus, the ampulla, and the infundibulum at the lateral fimbriated end [26] (Fig. 1).

### Tubular/sausage-shaped mass

**Fig. 2:**
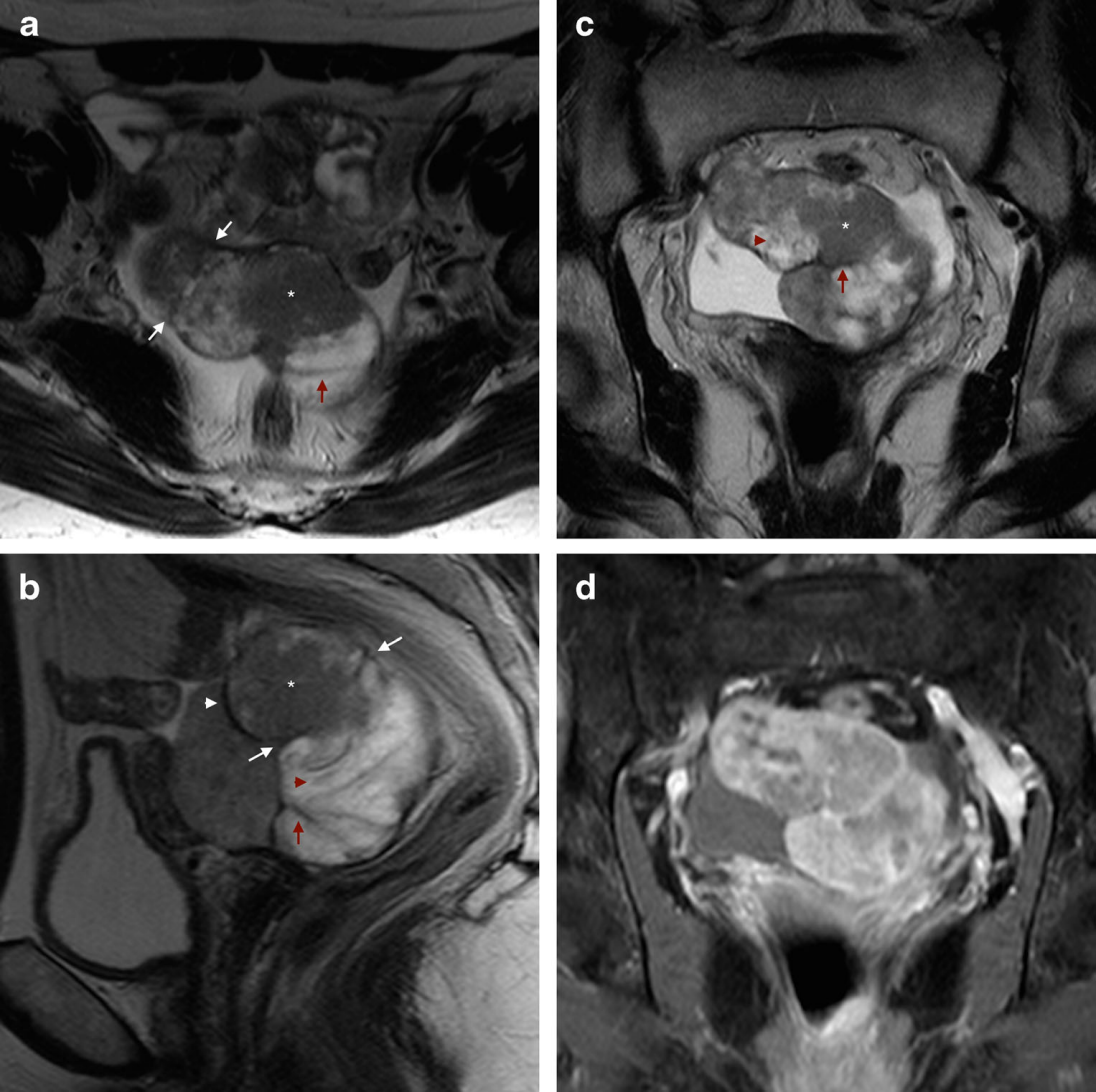
Serous PFTC in a 63-year-old female, presenting with pelvic pain. **a** Axial T2WI through the pelvis, showing “sausage”-shaped mass (*asterisk*). Incomplete folds (*red arrow*) and the “waist” sign (*white arrows*), are in favour of a fallopian tube mass; **b** Sagittal T2WI with the same solid mass (*asterisk*), inside a structure with well-defined walls (*white arrowhead*), the “waist” sign (*white arrows*), incomplete folds (*red arrow*), and the “synechiae” sign (*red arrowhead*) with strands of tissue floating in the fluid-filled fallopian tube; **c** Coronal T2WI, with the “sausage”-shaped mass (*asterisk*) folded upon itself, as depicted by the portions of wall visible inside the mass (*red arrow*), which approximates the “spoke-wheel” sign, usually better depicted when the mass is predominantly cystic. Once again, the “synechiae” sign can be observed (*red arrowhead*); **d** T1WI, fat-saturated, post-contrast image, showing the enhancement of the solid portions of the mass. Courtesy of Dra. Rosana Santos

The tubular nature of these paired organs is seen behind the sausage-shaped appearance when they are filled with solid tumour (Fig. 2). This is one of the most specific signs seen in PFTC, particularly in the presence of contrast enhancement of the mass (Figs. 2d and 5d). The solid tumour may have variable T1 hypointensity and T2 hyperintensity and show a high signal in diffusion weighted imaging (DWI) (Fig. 5). Other pathologies can present with hydrosalpinx and solid component, such as a tubo-ovarian abscess, and differentiation from malignancy can be challenging.

### Hydrosalpinx

**Fig. 3:**
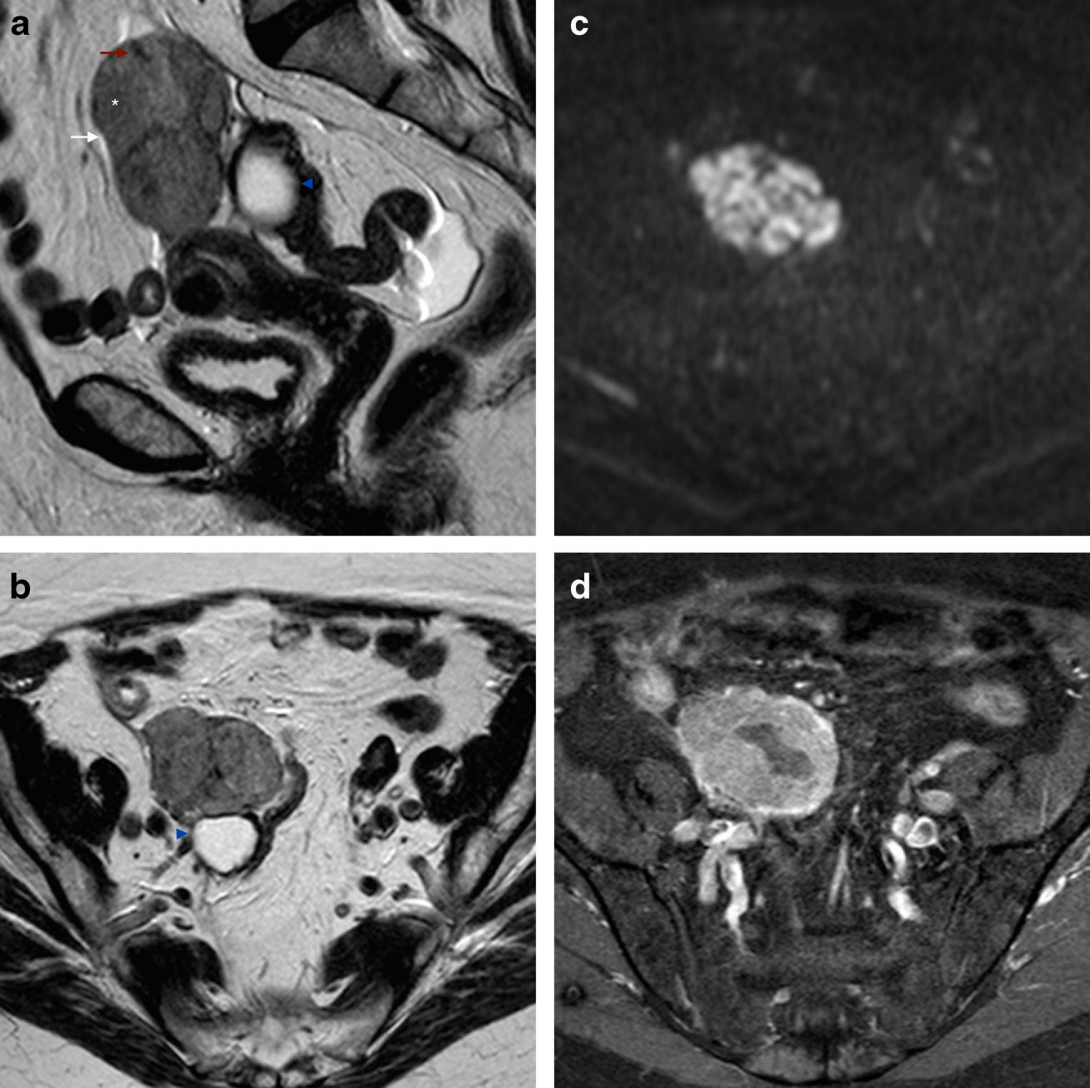
Serous PFTC in a 78-year-old female, presenting with postmenopausal bleeding and pelvic pain. **a** Sagittal T2WI showing a solid mass (*asterisk*) with well-defined walls (*white arrow*), incomplete folds (*red arrow*), and an incidental cystic lesion corresponding to a cystadenofibroma (*blue arrowhead*); **b** Axial T2WI showing the ovarian cystadenofibroma, posterior to the mass (*blue arrowhead*); **c** Axial DWI with the same solid mass showing restriction to diffusion; **d** Axial T1WI, fat-saturated, contrast-enhanced image, showing contrast uptake by the mass, with central necrosis

**Fig. 4:**
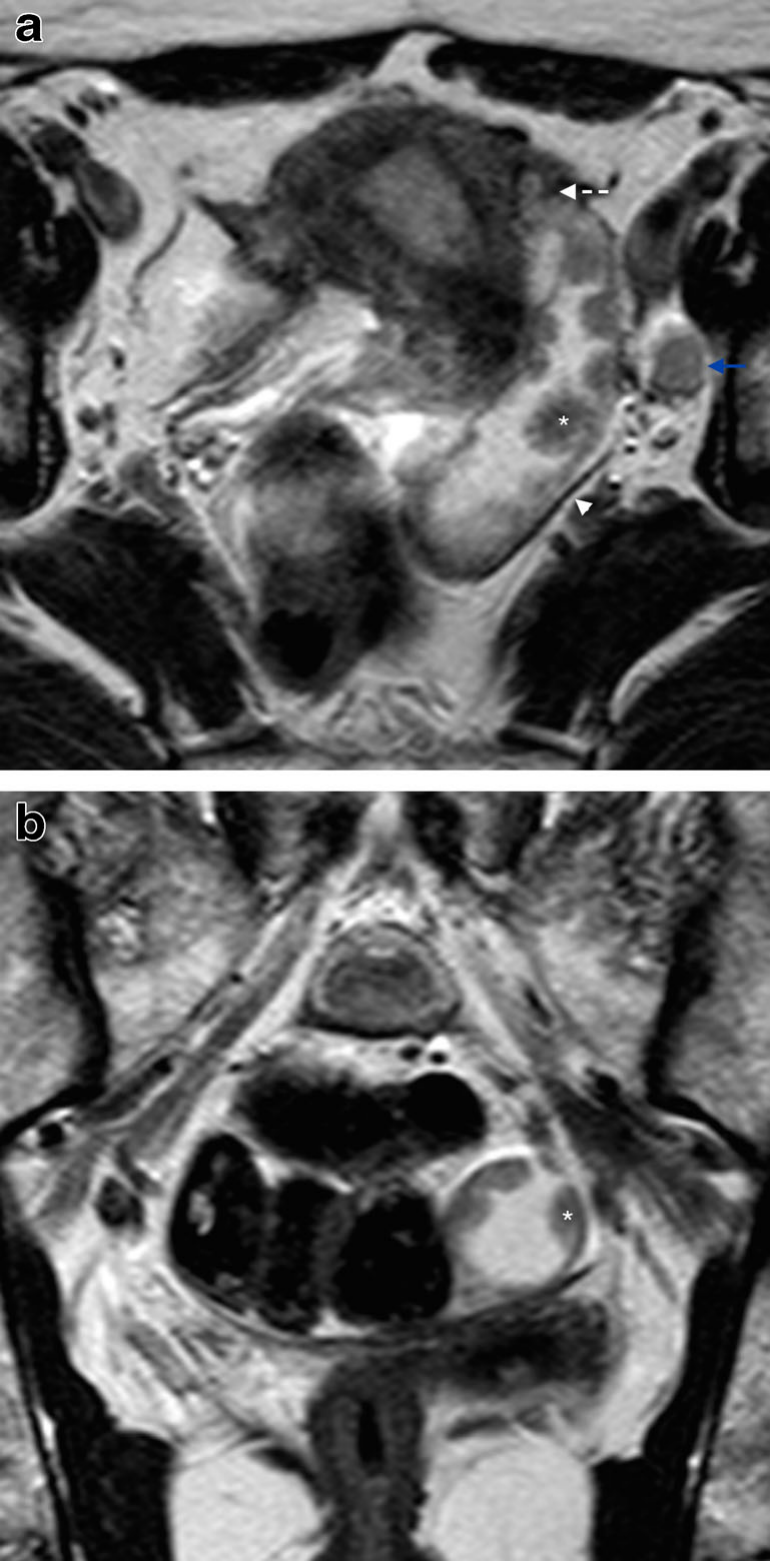
Serous PFTC in a 58-year-old female, presenting with postmenopausal bleeding. **a** Axial T2WI showing a left hydrosalpinx with well-defined walls (*arrowhead*), mural nodules (*asterisk*), the “beak sign” (*dashed arrow*), and an enlarged left obturator lymph node (*blue arrow*); **b** Coronal T2WI showing the same mural nodules (*asterisk*) inside the fluid-distended left fallopian tube, in transverse section

**Fig. 5:**
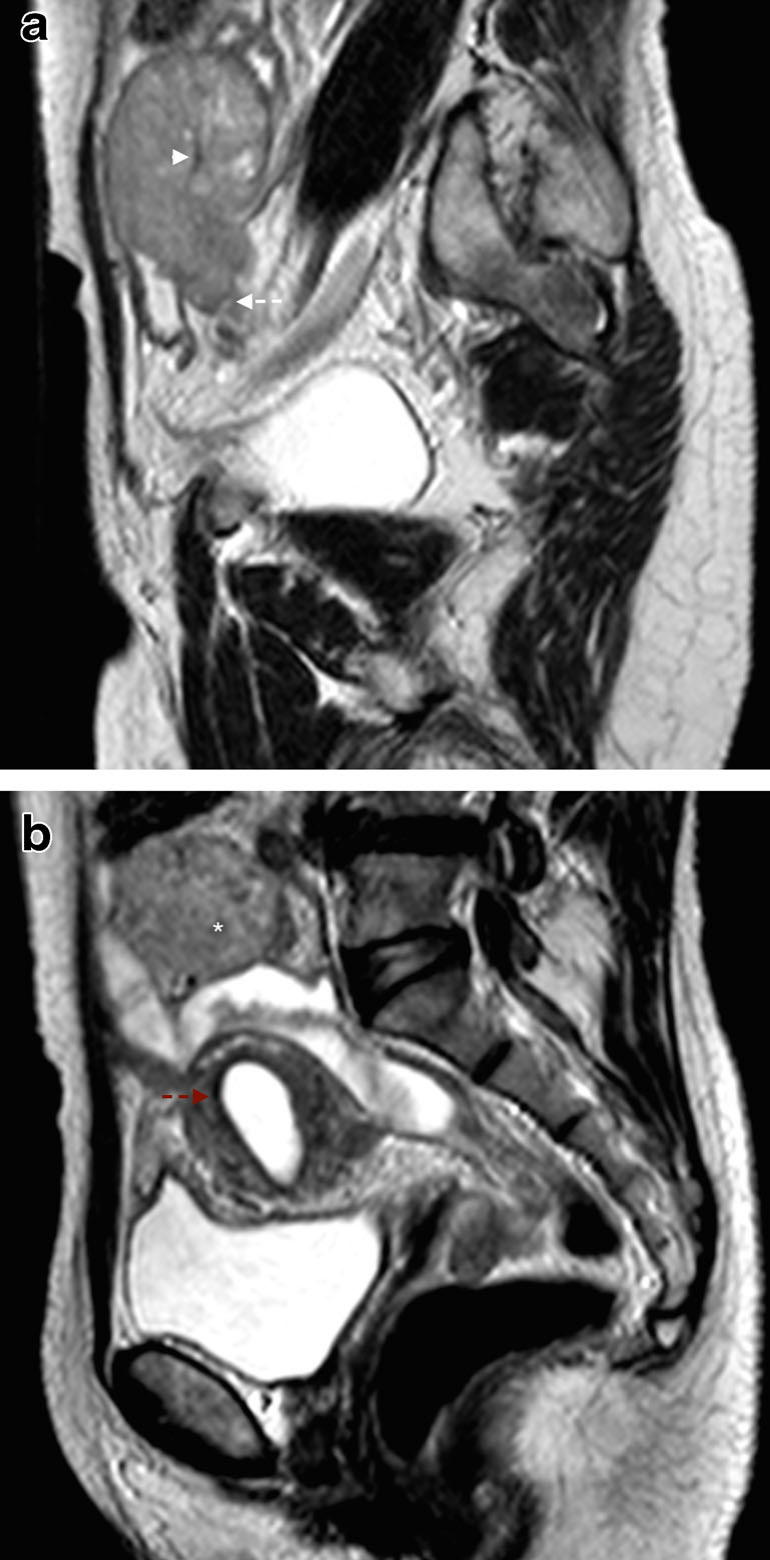
Serous PFTC in a 64-year-old female, presenting with abdominal pain. **a** Sagittal T2WI showing a “sausage”-shaped solid mass, folded upon itself as shown by the portion of Fallopian tube wall on the centre of the mass (*arrowhead*), and again the “beak” sign is depicted (*dashed arrow*); **b** Sagittal T2WI where the mass can still be seen (*asterisk*), together with an important sign in PFTC: hydrometra (*red dashed arrow*)

Hydrosalpinx forms in PFTC both due to the copious amounts of fluid produced by the tumour and to the partial obstruction of the tubes. This leads to tubular distention and can be easily observed in T2WI sequences. Frequently, one of the ends of the tube is patent, leading to decompression of the tubal obstruction with discharge and consequent shrinkage of the pelvic mass, resulting in a temporary resolution of symptoms, as described in the clinical presentation. This variability of size and shape of the mass can be seen in serial imaging. MRI can easily detect the presence of hydrosalpinx, which usually appears as a cystic, tubular-shaped, convoluted mass, with well-defined walls. The dilated tubes contain incomplete plicae or folds, producing its convoluted appearance, either seen as the “waist sign” (Fig. 2), causing focal constriction of the tubular structure, or as the “beak sign” (Figs. 3a and 4a), reflecting an acute angular contour, which would not be seen on a regular tubular or round structure [25]. This is distinct from the “beak sign” described as an indicative feature of an intra-ovarian lesion [54]. When a fallopian tube is extremely dilated by fluid, it may fold upon itself to produce the “cogwheel sign”, which may be indistinguishable from an ovarian neoplasm [2]. Occasionally, the “synechiae sign”, consisting of fine strands running across the lumen, can also be seen in dilated fallopian tubes [25] (Fig. 2b). Another MRI sign associated with the fallopian tubes is the “amorphous shading sign”, corresponding to the loss of signal intensity of the fluid filled tubes from T1WI to T2WI. The high viscosity and the presence of protein and iron due to recurrent haemorrhage, similar to what is seen in ovarian lesions, can explain this phenomenon [55]. The patency of the fallopian tube can lead to discharge of fluid into either the intra-uterine or the peritoneal cavities. However, ascites is not a specific sign of PFTC and is frequently present in ovarian cancer. Intra-uterine fluid, on the other hand, is specific of PFTC and it can occur in up to 30 % of cases [48] (Figs. 4b and 5).

## Conclusions

PFTC can be suspected on MRI when specific fallopian-tube related signs are present. However, the likelihood to present when already spread to the ovaries or peritoneum makes this diagnosis challenging, even for the experienced radiologist.

PFTC has a variety of MRI findings, which make it difficult to suspect its origin based solely on imaging. However, radiologists can be more suspicious in the presence of features related to fallopian tube disease, such as a relatively small, tubular-shaped (or sausage-shaped) mass, with homogenous signal, low signal intensity on T1WI, high signal intensity on T2WI, with mild to moderate enhancement, and associated hydrosalpinx or intrauterine fluid.

Singh’s [40] proposal for the histopathological diagnosis of high grade serous carcinoma, suggests assigning the fallopian tube as the primary site whenever there is a fallopian tube mass, even in the presence of other larger concomitant tumours. Radiologists should feel more confident in suspecting PFTC, and reporting it on their differential, in the presence of an adnexal mass associated to one or more of the three more specific signs (tubular-shaped mass, hydrosalpinx, and intrauterine fluid accumulation), particularly in earlier stage disease and when the ovaries can be identified as normal.

## Abbreviations

PFTC: Primary Fallopian Tube Carcinoma
FIGO: International Federation of Gynecology and Obstetrics
MRI: Magnetic Resonance Imaging
CT: Computed Tomography
EOC: Epithelial Ovarian Cancer
T1WI: T1 Weighted Images
T2WI: T2 Weighted Images
DWI: Diffusion Weighted Images
